# Useful Medicinal Plants for Vision Impairment in Traditional Iranian Medicine

**DOI:** 10.31661/gmj.v8i0.1285

**Published:** 2019-09-18

**Authors:** Jamshid Shayanfar, Hassan Ghasemi, Seyed Saeed Esmaili, Fatemeh Alijaniha, Ali Davati

**Affiliations:** ^1^Department of Iranian Traditional Medicine, School of Medicine, Shahed University, Tehran, Iran; ^2^Department of Ophthalmology, Shahed University, Tehran, Iran; ^3^Department of Traditional Medicine, School of Medicine, Shahed University, Tehran, Iran; ^4^Traditional Medicine Clinical Trial Research Center, Shahed University, Tehran, Iran; ^5^Department of Social Medicine, School of Medicine, Shahed University, Tehran, Iran

**Keywords:** Vision Impairment, Ophthalmology, Medicinal Plants

## Abstract

Vision impairment is an important general health issue that imposes many costs on governments and the health system every year. Despite the decline in infectious eye diseases, which has reduced the vision impairment and blindness over the past two decades, vision impairment is still a major health problem in some parts of the world. In traditional medicine books, visual weakness is referred to as "*any disturbance in the act of seeing* ". Many medicinal herbs have been mentioned in books of Traditional Iranian medicine (TIM) for the management of vision impairment. The aim of this study is to review the medicinal plants mentioned in TIM, which are considered effective for the treatment of vision impairment or its enhancement. In this library-based study, medicinal plants effective in the treatment of vision impairment were searched using 6 valid sources of traditional medicine, including *Makhzan ol-Adawiya*, *The Canon of Medicine*, *Tuhfat al-Momenin*, *Al-Abniyah An Haqaiq al-adwiya*, *Al-Shamil Fi al-Sana’at al-tebiyah*, and *Ekhtiarate Badiee*. This was done in 10 steps (finding keywords, searching for resources, preparing a single list, finding synonyms, classifying, reviewing, extracting plants from compositions, summarizing, scoring and sorting based on the obtained score). A total of 89 medicinal plants were extracted, most of which had a hot and dry temperament. Based on the obtained score, 12 plants got the highest scores (10 and above). The extracted plants can be the basis for further clinical studies to make new effective drugs for the prevention and treatment of vision impairment.

## Introduction

Visual impairment is a type of non-communicable disease that has mental and physical effects in the elderly. In 2010, 0.5% of the world population was blind, and 2.8% had severe-to-moderate vision impairment. In total, the major causes of vision impairment include, cataract, age-related macular degeneration (ARMD), uncorrected refractive errors, glaucoma, and corneal opacity [1]. Low vision refers to a condition where one is not blind, but has a less-than-normal vision. It is detected by a visual acuity of 3/60 to less than 6/18 in the healthier eye, after the best correction. Visual impairment also refers to conditions that range from low vision to blindness [2].In other words, low vision is a term for the vision impairments that cannot be corrected with standard eyeglasses or with medical or surgical treatments and may result from many eye or neurological diseases [3]. Studies have shown that vision impairment affects the quality of life by limiting social interactions and individual autonomy [4]. Visual impairment may appear with blind spots, decreased peripheral vision, decreased central vision, failure in contrast of the image, or the symptoms together. With aging and diseases such as diabetic retinopathy, the population with low vision has also increased [5]. The incidence of vision impairment also increases as the age increases among all age groups, and the risk of the disease in all regions of the world is higher in women than in men [6]. A study conducted in 2013 has estimated the financial cost of vision impairment in the United States adult persons at $51.4 billion per year [7]. In TIM references, weakness in vision has been considered as *Zafe Basereh*, and it has been defined as any disturbance in the act of seeing. In this disease, one cannot see the objects as they are even with effort, and an error occurs in the act of seeing [8].The approach of TIM toward health protection is based on improvement of lifestyle and emphasizes on the importance of prevention. In the area of ​​treatment, although pharmacotherapy and manipulation are used to treat patients, lifestyle modification, especially the emphasis on nutrition, has widened the horizons for researchers in a variety of diseases, including eye illnesses [9, 10]. Ancient Iranian physicians were experts in the field of diagnosis, description and treatment of eye diseases, as well as the definition of applied words. Almost all books of TIM have addressed the diagnosis and treatment of eye diseases. In addition, several specialized ophthalmological books have been written from the perspective of TIM [11]. About 25% of total prescribed drugs have been acquired from plants. Medicinal plants are famous for their little toxicity, effectiveness, and fewer side effects. A wide range of medicinal herbs has been recognized to be effective in complementary medicine schools, such as Ayurveda and Chinese medicine, in the treatment of eye diseases such as cataract and glaucoma [12]. Studies have shown that the probability of discovering a new effective drug increases up to 40% when traditional experiences are attended, in comparison to 1% in accidental researches. Thus searching in traditional textbooks may be an effective way for finding new drugs. [13]. This study aims to provide a classified and sorted list of medicinal plants mentioned in TIM references, which can be used for vision enhancement or treatment of vision weakness after conducting clinical studies.

## Search Strategis

This research is a library-based study and review. In this study, 6 important references of TIM have been used. Selected references have been chosen from various historical periods, including 1. *Al-Abniyah A nHaqaiq al-adwiya* (*Abu Mansour Movafagh Heravi* in the 4th century; *Hijri*), 2. *The Canon of Medicine* (*Ibn Sina* in the 5th century; *Hijri*), 3. *Al-Shamil Fi al-Sana’at al-tebiyah*(*Ibn Nafis* in the 7th century; *Hijri*), 4. *Ekhtiarate Badiee* (*Haji Zeiniddin Ali ibn Hussein Ansari* in the 8th century; *Hijri*), 5. *Tuhfat al-Momenin* (*MomenTonekaboni* in the 11th century; *Hijri)*, and 6. *Makhzan al-Adwiya* (*Mohammad Hussein Aghili Khorasani* in the 12th century; *Hijri*).Key-words including “eye” (with other synonyms in Arabic such as “*ain”*, and “*basar”),* and vision (with its synonym “*basereh”),* in addition toenhancement, strength, weakness, acuity, and clearing, were searched in the above-mentioned books. Subsequently, the selected plants that were effective in treating vision weakness or vision enhancement, were scored based on the model presented in the study of *Mozafarpour et al*. Using this model, he chose the medicinal plants effective for constipation and bloating according to TIM books [14, 15]. This scoring pattern was also used for other diseases such as palpitation [13]. In this model, scoring was done based on words indicative of the intensity and level of the effect on vision impairment or vision enhancement and sum of the scores in different books. The medicine with a stronger effect achieved a higher score, and the one with the weaker effect owned a lower score. This was done in 10 steps (finding keywords, searching for references, preparing a single list, finding synonyms, classifying, reviewing, extracting single herbs from compounds, summarizing, scoring, and sorting based on the obtained score). The plants were selected and classified if they had therapeutic properties rendering them useful for vision enhancement, acuity and clearing, or vision weakness. This pattern of defining criteria, scoring and sorting can create a categorized list of plants for researchers and provides the opportunity to choose other medicinal plants that are unavailable and, maladapted according to the patient’s situation (Table-1 and 2). Ranking of plants for clinical use has other considerations like less side effects and inexpensiveness which can change the rank of the plant in the list [16]. In addition to specific plants, the general influence on the eye health was also studied. Therout of administration and temperaments of each plant were also explained. In addition the positive effect of some medicinal plants on vision weakness (with higher score) in clinical studies, has been explained in new literature. The types of medicinal effects on vision weakness or enhancement have been evaluated.

## Results

After collecting and scoring the plants, a total of 89 plants ( Table-3) were found to have an effect on vision enhancement or weakness which sorted from stronger effect to weaker [17-22]. A vast majority of the plants had a dry temperament, and most of them were hot and dry (Table-4).A total of 12 plants *Hasha, Hozoz, Harmal, Balsan, Farasiun, Esghil, Sodab, Razianaj, Basal, Balilaj, Heltit, and Enab-o-Salab*, scored higher than other plants. Most of the plants were extracted from *Makhzan al-Adwiya*, and the least ones were compiled from *Al-AbniyahAnHaqaiq al-adwiya* (Table-5). Most plants were found to be effective in vision enhancement, and a smaller number on vision impairment (Table-5).The rout of administration for most plants was similar to *Kohl* (Collyrium), and for a smaller number, it was oral.The results of clinical studies searched for plants with higher score (10 and above) are as follows: The aqueous extract of the seeds of *Razianaj* (*Foeniculum vulgare)* has protective and therapeutic properties against sodium selenite-induced cataract in rabbit’s eye. The possible mechanism of this extract is antioxidant activity. Different phytochemical studies have shown the presence of compounds like flavonoids, phenolic compounds, and volatile compounds, in this plant. It has several other pharmacological properties such as anti-inflammatory, oculohypotensive effects. Lens opacity scores were lower in rabbits that received aqueous extract eye drops of this plant twice daily for 5 days before cataract induction and 21 days after cataract induction[23]. Another study by Agarwal *et al*. showed that *Foeniculum vulgare* aqueous extract reduces intraocular pressure and the maximum reduction in pressure is comparable with timolol. Its mechanism of action is through an anti-cholinesterase effect [24].Sudhakar *et al*. showed that the oral use of *Sodab* (*Ruta graveolens*) two times a day for 2 years clearly reduced the annual progression of myopia, and even after the discontinuation of the medication, the speed of progression of myopia decreased. The mechanism of action of this plant is likely through the ciliary body, which leads to the exact focusing of the image on the retina by improving the accommodation in myopic individuals even while working near the object [25]. Javadzadeh *et al*. presented that the administration of 1 drop of onion extract in mice eyes for 14 days every 8 hours prevents the formation of selenite-induced cataract [26]. In 2010, a study conducted by Gupta *et al.* on 9-day-old rat pups showed that intraperitoneal injection of the aqueous extract of Triphala (combination of *Emblica officinalis*, *Terminalia bellerica,* and *Termina liachebula*) at doses of 25mg/kg body weight prevents selenite-induced cataract formation the formation of selenite-induced cataract. In addition, it enhances the activity of antioxidant enzymes, glutathione, and decreases the extent of lipid peroxide in contradiction of selenite-induced oxidative stress [27]. Another case-control study has proved the role of oxidative stress as the main mechanism of eye damage in cataract, glaucoma, and ARMD, suggesting the role of plasma antioxidant capacity in preventing these eye diseases. Plasma antioxidant capacity increases with foods rich in antioxidants, like fruits and vegetables [28]. Despite the searches about plants with higher score, sufficient evidence of the effectiveness of all plants on vision weakness extracted from the references was not found, but the antioxidant properties of many of these plants, suggest their potency in the treatment of vision impairment or its enhancement. These include *Solanum nigrum* [29] *,Peganum harmala* [30], *Zingiber officinalis* [31], *Ferula assa- foetida* [32], *Rheum ribes* [33] ,and *Terminalia bellerica* [34] .

## Discussion

The causes of vision impairment in TIM include bodily causes, brain causes, causes specific to vision power, causes related to the eye layers, and causes related to the nerve that should be considered in the treatment of vision impairment Ancient Iranian physicians addressed the primary cause of the disease while paying attention to 6 essential principles of maintaining health (climatic conditions and environment, food and drink, physical activities and rest, evacuation and retention, psychiatric conditions, sleep and wakefulness), and finally, they used medications and manipulation or physical therapy (such as surgery).For example, in cataract (*Nozulol-ma*), Ancient Iranian physicians believed that in the early stages of the disease, treatment is possible through nutrition and drug therapy, but when the disease progresses, surgery is the only treatment. Patients are advised to avoid eating meat, fish, and fruits, and instead, consume dry foods. In the *Canon of medicine, Avicenna* emphasizes that eye probing for cataract surgery should not be done quickly, and should be waited for until its completion [35]. Some inappropriate and harmful conditions and foods lead to vision weakness and should be avoided from TIM point of view.These are: dust, smoke, very hot or very cold weather, continuous gaze to objects specially very small things, too much crying, too much intercourse, exceed drinking alcohol, overeating and sleeping immediately after eating food, eating excess salt, eating foods like lentil, cabbage, garlic, sugar, long or short sleeping, and prolonged watching luculent objects. Some useful plants for eyesight are eating foods in moderate amounts, moderate sleeping, eating cinnamon, saffron, star anise and turnips [22]. The main mechanism of eye damage in cataract, glaucoma, and ARMD is oxidative stress. Antioxidants form the first layer of defense in contrast to oxidative stress and are got with diet and internal production. The most important antioxidants that perform a protective role in the eyes include ascorbic acid, superoxide dismutase catalase, and reduced glutathione. Initial studies suggest that the antioxidant capacity of the plasma can increase with diets rich in antioxidants, like fruits and vegetables, through the diet [28].In addition, there is evidence that oxidative stress leads to the activation of the molecular components contributing to the development and appearance of diseases associated with myopia [36]. A wide range of medicinal herbs is used in the management of some eye diseases, including cataract and glaucoma. Although in some health systems such as Ayurveda and Traditional Chinese Medicine, common therapeutic properties have been expressed for a plant, in the treatment or prevention of the vision impairment has been shown in a few clinical studies in modern medicine. Considering the 6 principles of maintaining health, and preventive effects of herbal medicine in early stages, TIM emphasizes on vision enhancement against vision weakness and health- maintaining principles rather than the disease treatment alone. Obviously, in advanced stages of the disease, surgery will be more important than other methods, and herbal medicines may be considered as a complementary treatments along with the surgery.

## Conclusion

According to TIM and studies conducted, many medicinal herbs can be used in the treatment of vision impairment, and it is necessary to conduct more precise clinical and animal studies about the widespread use of these plants in its treatment, and this study can be the first step in this direction.

## Conflict of Interest

None.

**Table 1:** The Criteria to Score Properties Mentioned for the Plants in the Books

A strong emphasis on the vision enhancement or the effectiveness on vision impairment with terms such as seriously, strongly beneficial, experimented, ultimately, and intense.	3
The expression of the vision enhancement or effectiveness on vision weakness with terms such as usefulness for vision weakness or vision enhancement, and vision sharpening	2
Expressing the effects on eye health with terms such as usefulness or beneficial for eyes The implicit expression of vision enhancement or the effectiveness on vision weakness with terms such as eliminating vision darkness, clearing vision, and increasing the light of eye.	1

**Table 2:** A Scoring Example of the Plants

**Book** **Plant**	***Makhzan*** *** al-*** ***Adwiya***	***the Canon of medicine***	***Al-*** ***Abniyah*** *** An *** ***Haqaiq*** *** al-*** ***adwiya***	***Al-*** ***Shamil*** *** Fi al-*** ***Sana’at*** *** al-*** ***tebiyah***	***Tuhfat*** *** al-*** ***Momenin***	***Ekhtiarate*** ***Badiee***
**Hasha**	Suitable for vision weakness and its enhancement	Maintains the power of vision and eliminates vision darkness	Useful for vision weakness	Subtilizes the eye and enhances it strongly	Eating a small amount with food is useful for vision enhancement	It is useful for vision weakness and maintains its enhancement
**Score**	2	2	2	3	2	2
**Total score**	13					
**Mamiran**	Using as kohl is useful for vision darkness	Sharpens the vision with its use as kohl	Sharpens the vision	Using as kohl sharpens the vision, thereby enhancing it	Using as kohl is useful for vision darkness	Increases the light of eye
**Score**	1	2	2	2	1	1
**Total score**	9					

**Table 3:** The Effective Medicinal Plants in the Treatment of Vision Weakness

**Traditional name**	**Scientific name**	**Temperament**	**Rout of administration**	**Score**	**Ref. No**
*Hasha*	*Thymus capitatus*	Hot and dry	Oral	13	1-6
*Hozoz*	*Lycium afrum L.*	Moderate and dry	Using as *kohl*	12	1-6
*Harmal*	*Peganum harmala L.*	Hot and dry	Using as *kohl*	12	1-6
*Balsan*	*Commiphora gileadensis*	Hot and dry	Using as *kohl*	12	1-6
*Farasiun*	*Marrubium vulgare L.*	Hot and dry	Suppository-eye drop	12	1-6
*Esghil*	*Scilla maritima L.*	Hot and dry	Oral	11	2-6
*Sodab*	*Ruta graveolens L.*	Hot and dry	Oral	11	1, 2, 4-6
*Razianaj*	*Foeniculum vulgare L.*	Hot and dry	Using as *kohl*	10	1-6
*Basal*	*Allium cepa L.*	Hot and dry	Oral	10	1-4,6^1^-^4^, ^6^
*Balilaj*	*Terminalia bellerica*	Cold and dry	Oral	10	2-6
*Heltit*	*Ferula assa-foetida L.*	Hot and dry	Using as *kohl*	10	3-6
*Enab-o-Salab*	*Solanum nigrum L.*	Cold and dry	Using as *kohl*	10	2-6
*Oshnah*	*Useneabar bata Ach*	Moderate	Using as* kohl*	9	2-6
*Darsini*	*Cinnamomum zeylanicum*	Hot and dry	Using as* kohl*	9	1-6
*Ribas*	*Rheum ribes L.*	Cold and dry	Using as *kohl*	9	2-6
*Sokar-ol-Oshr*	*Calotropis proceradryand*	Hot and dry	Using as *kohl*	9	2-6
*Sabr*	*Aloe vera L.*	Hot and dry	Using as* kohl*	9	1, 3-6
*Satar*	*Thymus vulgaris*	Hot and dry	Oral	9	1, 3-6
*Gharanfol*	*Eugenia caryophyllata*	Hot and dry	Using as *kohl*	9	2-6
*Mamiran*	*Chelidonium majus L.*	Hot and dry	Using as* kohl*	9	1-6
*Ostokhodus*	*Lavandula stoechas L.*	Hot and dry	Oral	8	3, 5, 6
*Amolaj*	*Phyllanthus emblicaL.*	Cold and dry	Oral	8	2, 3, 5, 6
*Ejas*	*Prunus domestica L.*	Cold and wet	Using as* kohl*	8	2, 3, 5, 6
*Javshir*	*Opopanax chironium/ (L.)Koch.*	Hot and dry	Using as *kohl*	8	2-4, 6
*Khelaf*	*Salix alba L.*	Cold and dry	Using as *kohl*	8	2-4, 6
*Roman*	*Tunica granatum*	Cold and wet	Using as* kohl*	8	3-6
*Loz-ol-Mor*	*Amygdalus amara Hayne*	Cold and dry	Using as *kohl*	8	2-4, 6
*Kornab*	*Brassica oleracea L.*	Hot and dry	Oral	8	2-6
*Mamitha*	*Glaucium corniculatum L*	Cold and dry	Using as *kohl*	8	3-6
*Mor*	*Commiphora* *Myrrha(Nees)*	Hot and dry	Using as* kohl*	8	3-6
*Aas*	*Myrtus communis L.*	Cold and dry	Using as* kohl*	7	4-6
*Aghaghia*	*Acacia arabica*	Cold and dry	Using as *kohl*	7	2-4, 6
*Athl*	*Tamarix gallica L.*	Cold and dry	Eye drop	7	3, 5, 6
*Badruj*	*Ocimum basilicum L.*	Hot and dry	Eye drop	7	2-6
*Bokhur-e-Maryam*	*Cyclamen europium L.*	Hot and dry	Using as* kohl*	7	1, 3, 4
*Tormes*	*Lupinus angustifoliusL.*	Hot and dry	Oral	7	3, 5, 6
*Salikheh*	*Cinnamomum bejolghota (Buch. /Ham.) Sweet*	Hot and dry	Using as* kohl*	7	1,3-6
*Sakbinaj*	*Foeul aperica*	Hot and dry	Using as *kohl*	7	2-5
*Shaljam*	*Brassica rapa L.*	Hot and wet	Oral	7	2,3,5,6
*Felfel*	*Piper nigrum L.*	Hot and dry	Using as *kohl*	7	2-6
*Ghasab-ol-Zarirah*	*Arundo phragmites L.*	Hot and dry	Using as *kohl*	7	2,3,5,6
*Ahlilaj-e-Asfar*	*Terminalia citrina Roxb.*	Cold and dry	Using as *kohl*	6	2,3,5,6
*Joze-Bova*	*Myristica fragrans L.*	Hot and dry	Using as *kohl*	6	2,5,6
*Kharbagh-e-Asvad*	*Helleborus niger L.*	Hot and dry	Using as *kohl*	6	2,4,6
*Dam-ol-Akhavein*	*Dracaena cinnabari Balf.f.*	Cold and dry	Using as *kohl*	6	4-6
*Zaferan*	*Crocus sativus L.*	Hot and dry	Using as *kohl*	6	2-6
*Somagh*	*Rhus coriaria L.*	Cold and dry	Using as *kohl*	6	3,5,6
*Sonbol*	*Hyacinthus orientalis*	Hot and dry	Using as* kohl*	6	3,5,6
*Shaghayegh*	*Anemone sp.*	Hot and dry	Using as *kohl*	6	2-4,6
*Shahtareh*	*Fumaria parviflora Lam.*	Moderate and dry	Using as *kohl*	6	3,5,6
*Tabashir*	*Bambusa arundinacea Willd*	Cold and dry	Nasal snuff	6	5,6
*Kondosh*	*Gypsophila struthium L.*	Hot and dry	Nasal snuff	6	3,5,6
*Marzanjush*	*Origanum majorana L.*	Hot and dry	Using as* kohl*	6	3,5,6
*Vaj*	*Acorus calamus L.*	Hot and dry	Using as *kohl*	6	1-3,5,6
*Hesrem*	*Vitis vinifera L.*	Cold and dry	Using as *kohl*	6	3,6
*Kharbagh-e-Abyaz*	*Veratrum album L.*	Hot and dry	Using as *kohl*	5	2,4,6
*Zanjebil*	*Zingiber officinale roscore*	Hot and dry	Using as *kohl*	5	1-4
*Gharasia*	*Pranus avium L.*	Cold and dry	Using as* kohl*	5	4-6
*Oqhovan*	*Tanacetum parthenium*	Hot and dry	Using as* kohl*	4	5,6
*Barsian*	*Albizzia lebbeck (L.) Bth.*	Hot and dry	Oral- Smelling	4	3,6
*Tashmizaj*	*Cassia absus*	Hot and dry	Using as *kohl*	4	5,6
*Jadvar*	*Curcuma zedoaria Rosc.*	Hot and dry	Eye drop	4	5,6
*Khardal*	*Brassica juncea (L.) Czern*	Hot and dry	Using as *kohl*	4	3,6
*Dardar*	*Ulmus campestris*	Cold and dry	Using as* kohl*	4	2,4-6
*Sous*	*Glycyrrhiza glabra L.*	Hot and dry	Using as* kohl*	4	5,6
*Selgh*	*Beta vulgaris L.*	Hot and dry	Using as* kohl*	4	1,3
*Gharab*	*Salix babilonica L.*	Cold and dry	Using as *kohl*	4	2,4-6
*Fotr*	*Funji spp*	Cold and wet	Using as *kohl*	4	5,6
*Kravia*	*Cuminum cyminum L.*	Hot and dry	Using as* kohl*	4	2,6
*Komon*	*Carum carvi L.*	Hot and dry	Kohl-Eye drop	4	2,3,6
*Kondor*	*Boswellia carterii*	Hot and dry	Using as kohl	4	3,5,6
*Mash*	*Vigna radiata R.wilczek*	Cold and dry	Oral	4	5,6
*Anbarbaris*	*Berberis vulgari L.*	Cold and dry	Using as *kohl*	3	3
*Anison*	*Pimpinella anisum L*	Hot and dry	Using as *kohl*	3	3
*Zaitun*	*Olea europaea L.*	Hot and dry	Using as *kohl*	3	2,4
*Sokkar*	*Saccharum officinarum L.*	Hot and dry	Using as *kohl*	3	2,3,5
*Ghatran*	*Pix liquida*	Hot and dry	Using as* kohl*	3	2,4
*Kafur*	*Laurus camphora*	Cold and dry	Using as *kohl*	3	1,3
*Asarun*	*Asarum europaeum L*	Hot and dry	Oral	2	3
*Anzarut*	*Astragalus sarcocolla Dym*	Hot and dry	Using as *kohl*	2	3
*Otroj*	*Citrus medicavar.cedrata*	Cold and dry	Using as *kohl*	2	3
*Afsantin*	*Artemisia absinthium L.*	Hot and dry	Using as *kohl*	2	3
*Bohnkareh*	*Ealipta alba*	Hot and dry	Oral	2	6
*Hur*	*Populus alba*	Hot and dry	Using as *kohl*	2	2
*Zaravand*	*Aristolochia longa L.*	Hot and dry	Oral	2	3
*Faranjmeshk*	*Ocimum pilosum*	Hot and dry	Apply on head	2	3
*Ghantorion*	*Centaurea cyanus L.*	Hot and dry	Using as *kohl*	2	3
*Limun*	*Citrus aurantifolia L.*	Cold and dry	Using as *kohl*	2	3
*Kam-at*	*Tuber album Sow.*	Cold and wet	Using as *kohl*	2	2,3

**Table 4:** The frequency of plants natures

**Temperament**	**Hot and dry**	**Cold and dry**	**Cold and wet**	**Moderate and dry**	**Hot and wet**	**Moderate**
Number	58	23	4	2	1	1

**Table 5:** The Frequency of Effective Medications and Usefulness of Medicinal Plants on Vision Weakness or Enhancement

** Book** **No or effectiveness**	***Makhzan*** *** al-*** ***Adwiya***	***the Canon of medicine***	***Al-*** ***Abniyah*** *** An *** ***Haqaiq*** *** al-*** ***adwiya***	***Al-*** ***Shamil*** *** Fi al-*** ***Sana’at*** *** al-*** ***tebiyah***	***Tuhfat*** *** al-*** ***Momenin***	***Ekhtiarate*** ***Badiee***
**Number of selected plants**	69	46	18	67	58	43
**Effectiveness on vision enhancement**	42	11	4	29	32	11
**Effectiveness on vision weakness**	8	4	2	2	9	4
